# Bread Feeding Is a Robust and More Physiological Enteropathogen Administration Method Compared to Oral Gavage

**DOI:** 10.1128/IAI.00810-19

**Published:** 2020-03-23

**Authors:** Anne Derbise, Hebert Echenique-Rivera, Marta Garcia-Lopez, Rémi Beau, Myriam Mattei, Hugo Varet, Petra Dersch, Javier Pizarro-Cerdá

**Affiliations:** a Yersinia Research Unit, Institut Pasteur, Paris, France; b Animalerie Centrale, Centre de Ressources et Recherches Animales, Institut Pasteur, Paris, France; c Bioinformatics and Biostatistics Hub, Département Biologie Computationnelle, Institut Pasteur, USR 3756 CNRS, Paris, France; d Institute of Infectiology, Center for Molecular Biology of Inflammation, University of Münster, Münster, Germany; University of California San Diego School of Medicine

**Keywords:** oral infection, pathogenesis, bread feeding, *Yersinia pseudotuberculosis*, *Yersinia enterocolitica*, enteropathogen

## Abstract

Oral administration is a preferred model for studying infection by bacterial enteropathogens such as Yersinia spp. In the mouse model, the most frequent method for oral infection consists of oral gavage with a feeding needle directly introduced in the animal stomach via the esophagus. In this study, we compared needle gavage to bread feeding as an alternative mode of bacterial administration. Using bioluminescence-expressing strains of Yersinia pseudotuberculosis and Yersinia enterocolitica, we detected very early upon needle gavage a bioluminescent signal in the neck area together with a signal in the abdominal region, highlighting the presence of two independent sites of bacterial colonization and multiplication. Bacteria were often detected in the esophagus and trachea, as well as in the lymph nodes draining the salivary glands, suggesting that lesions made during needle introduction into the animal oral cavity lead to rapid bacterial draining to proximal lymph nodes. We then tested an alternative mode of bacterial administration using pieces of bread containing bacteria. Upon bread feeding infection, mice exhibited a stronger bioluminescent signal in the abdominal region than with needle gavage, and no signal was detected in the neck area. Moreover, Y. pseudotuberculosis incorporated in the bread is less susceptible to the acidic environment of the stomach and is therefore more efficient in causing intestinal infections. Based on our observations, bread feeding constitutes a natural and more efficient administration method which does not require specialized skills, is less traumatic for the animal, and results in diseases that more closely mimic foodborne intestinal infection.

## Abstract (precis)

Oral administration is a preferred model for studying infection by bacterial enteropathogens such as Yersinia spp. In the mouse model, the most frequent method for oral infection consists of oral gavage with a feeding needle directly introduced in the animal stomach via the esophagus. In this study, we compared needle gavage to bread feeding as an alternative mode of bacterial administration. Using bioluminescence-expressing strains of Yersinia pseudotuberculosis and Yersinia enterocolitica, we detected very early upon needle gavage a bioluminescent signal in the neck area together with a signal in the abdominal region, highlighting the presence of two independent sites of bacterial colonization and multiplication.

## INTRODUCTION

Animal infection models represent major research tools to understand human disease, as they allow one to investigate in a relevant physiological environment the very complex interactions that take place between pathogens and hosts during organ/tissue colonization or whole-body dissemination (1, 2). For the study of bacterial enteropathogens, oral administration is a preferred infection route to assess intestinal colonization and pathogenesis in mammalian hosts. The most often used methodology for oral infection in laboratories using mice as models consists of oral gavage with a feeding needle introduced in the stomach via the esophagus. However, several laboratories have reported, using bioluminescence-expressing pathogens, colonization in sites distant from the abdominal region after orogastric infection, suggesting abrasions in the laryngopharynx region (3–6). Although the impact of such accidental colonization on the overall infectious process is not known, we cannot exclude the possibility of bacterial dissemination in the bloodstream independently from the intestinal infection. In order to avoid this potential problem, alternative modes of pathogen administration have been described, such as bread feeding (7, 8) or drinking water delivery (9, 10).

Enteropathogenic Yersinia spp. are the third leading bacterial cause of human gastrointestinal infections in Europe, with Yersinia enterocolitica being the major agent responsible for this disease (11). Although isolated much less often than Y. enterocolitica, Yersinia pseudotuberculosis is responsible for acute gastroenteritis and mesenteric lymphadenitis in a wide variety of animals, including rodents, domestic animals, nonhuman primates, and humans (12). After oral ingestion, Y. pseudotuberculosis localizes to the ileum and proximal colon and can pass/cross the intestinal barrier, invading its host through gut-associated lymphoid tissues and Peyer’s patches (13). In humans, dissemination to deeper tissues and the bloodstream is frequent in patients with underlying disease conditions (such as diabetes mellitus, liver cirrhosis, and hemochromatosis) and can lead to septicemia as a severe outcome of the infection (14, 15).

While performing mouse oral administration of Y. pseudotuberculosis with a feeding needle, it has been regularly noticed that some animals exhibit spleen infections without displaying the presence of bacteria in Peyer’s patches (PPs) or mesenteric lymph nodes (MLNs), suggesting the passage of bacteria in the bloodstream independently of the colonization of the gut-associated lymphoid tissues (16). Since it has been reported that needle gavage can cause lesions in the oropharyngeal region (3), we decided to reexamine this mode of administration and compare it to an alternative administration method (bread feeding) with fully virulent Y. pseudotuberculosis and Y. enterocolitica strains expressing bioluminescence in order to follow bacterial dissemination over time in the whole animal body. Our results clearly illustrate that needle feeding promotes bacterial colonization not only of the intestinal tract but also the neck region with a tropism for salivary glands and lymph nodes. On the contrary, bread feeding induces a much more robust intestinal tract infection that is never associated with neck region infections. Moreover, bread feeding allows bacteria to be protected from the acidic environment of the stomach. We conclude, therefore, that bread feeding is a better oral administration route to investigate the pathophysiology of bacterial enteropathogen infection.

## RESULTS

### Construction of constitutively bioluminescent Y. pseudotuberculosis and Y. enterocolitica strains.

The IP32953 Y. pseudotuberculosis strain, isolated from the stools of a human patient, was chosen for this study due to its capacity to express two major virulence factors, the type 3 secretion system (T3SS) and the yersiniabactin (Ybt) iron uptake machinery. This strain has been previously shown to be fully virulent in laboratory mice (17, 18). The WA Y. enterocolitica strain belongs to biotype 1B serotype O:8, and it was isolated from the blood of a human patient and was shown to be virulent in rodents (19).

To perform a comparative analysis of bacterial dissemination *in vivo* after oral infection, strains IP32953 and WA were genetically engineered to constitutively express bioluminescence. In order to correlate bioluminescence to bacterial numbers during animal infection, the *luxCDABE* operon (placed under the control of the constitutive *rplN* promoter) was stably introduced into the *Yersinia* chromosome using the mini-Tn*7* transposon technology (20, 21).

The resulting strains, IP32953-*lux* and WA-*lux*, harbor the construct *mini-Tn7-Km-P_rplN_-luxCDABE* between the two housekeeping genes *glmS* and *pstS* (see Fig. S1A in the supplemental material) (21). The stability of the bioluminescence signal over time was determined by performing 10 subcultures of IP32953-*lux* in LB without antibiotics over 17 days. At different time points, bacterial aliquots were streaked on LB plates, and individual colonies were checked for their capacity to emit bioluminescence. After 10 subcultures, 100% of the CFU expressed bioluminescence, confirming the stability of the bioluminescent phenotype in the bacterial population.

The IP32953-*lux* and WA-*lux* strains displayed *in vitro* growth similar to that of the parental strains (Fig. S2). The IP32953-*lux* strain exhibits virulence similar to that of the parental wild-type IP32953 strain (Fig. S3). Therefore, IP32953-*lux* was used to determine the characteristics and relevant differences between the two infection methods applied, oral gavage with a feeding needle versus bread feeding. The WA-*lux* strain was used as a second species to confirm some of the results obtained with Y. pseudotuberculosis.

### Pathogenic *Yersinia* spp. colonize the neck and the abdominal region of infected animals upon needle feeding.

We first set up the bread feeding protocol in OF1 mice based on a previously reported methodology (7). In our study, instead of using melted butter as a vehicle to deliver bacteria and to attract mice to the bread, mice were first trained to feed on bread only (see Materials and Methods) and then were infected with bread supplemented with a bacterial suspension in phosphate-buffered saline (PBS). A pilot experiment using this new method allowed us to validate that within less than 5 min, mice consumed bread supplemented with 8E7 CFU of IP32953-*lux*, subsequently exhibiting bioluminescence in the PPs and MLNs, therefore confirming Y. pseudotuberculosis colonization and dissemination in the intestinal tract as expected for an enteropathogen (Fig. S1B).

**FIG 1:**
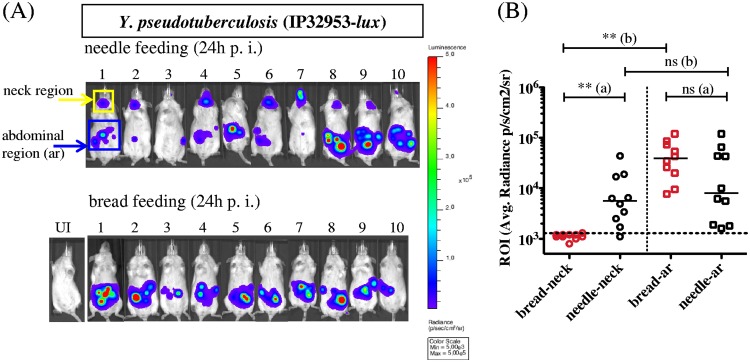
Comparative bioluminescence imaging of mice infected by either needle or bread feeding. OF1 mice were infected with 3.5E8 CFU of a Y. pseudotuberculosis bioluminescent strain (IP32953-*lux*) using a 20 G by 1.5” feeding needle or a piece of bread. (A) At 24 h postinfection (h p.i.), mice were imaged using an IVIS Spectrum system with an acquisition time of 2 min and small binning. Uninfected (UI) mice were used to set the light emission background. Regions of interest (ROI) were drawn in the neck region (yellow frame) and the abdominal region (ar; blue frame) using the Living Image 4.5 software. Min, minimum; Max, maximum. (B) ROI average (Avg.) bioluminescence (photons/s/cm^2^/sr) was calculated for each individual mouse infected by needle gavage (in black) or by bread feeding (in red). The horizontal dotted line indicates the light emission background. (a and b) Data were analyzed using the Prism 5.0 software for nonparametric Mann-Whitney *t* test (nonsignificant [ns], *P* > 0.05; **, *P* < 0.0015) (a), and for nonparametric Wilcoxon matched-pairs signed-rank test (ns, *P* > 0.05; **, *P* < 0.003) (b). The median of the values is indicated by a horizontal bar. Bioluminescence signal is detected in the neck of 80% of the needle-infected animals, with an average radiance not statistically different than the signal from the abdominal region, whereas none of the mice infected with bread presented a signal in the neck. Although the median of the abdominal region is higher when mice are infected with bread, the overall signal is not statistically different due to the variability between mice.

We then proceeded to compare needle versus bread feeding as oral administration routes. Twenty-four hours after oral administration of 3.5E8 CFU of IP32953-*lux*, mice were monitored for bioluminescence, and regions of interest (ROI) were identified for bioluminescence imaging (BLI) measurements. Upon needle gavage, up to 80% of the mice displayed a BLI signal in two distinct regions of the body, one in the expected abdominal region and a second in the neck, whereas upon bread feeding, none of the mice exhibited a neck signal, and bioluminescence was restricted to the abdomen (Fig. 1A). ROI measurements in the neck region of needle-infected mice indicate that the level of BLI is similar to that coming from the abdominal region, suggesting that bacteria colonize, disseminate, and multiply in the neck as efficiently as in the abdomen (Fig. 1B). This comparative analysis indicates that oral administration of Y. pseudotuberculosis using needle feeding results in infection of the neck region, a phenotype that is not observed upon bread feeding. A similar phenotype was observed using the Y. enterocolitica WA-*lux* strain, indicating that this phenomenon could be extended to other pathogens (Fig. S4).

### Upon needle feeding, pathogenic *Yersinia* spp. multiply in the intersection of the esophagus and trachea and disseminate to the draining lymph nodes of the submandibular area.

**FIG 2:**
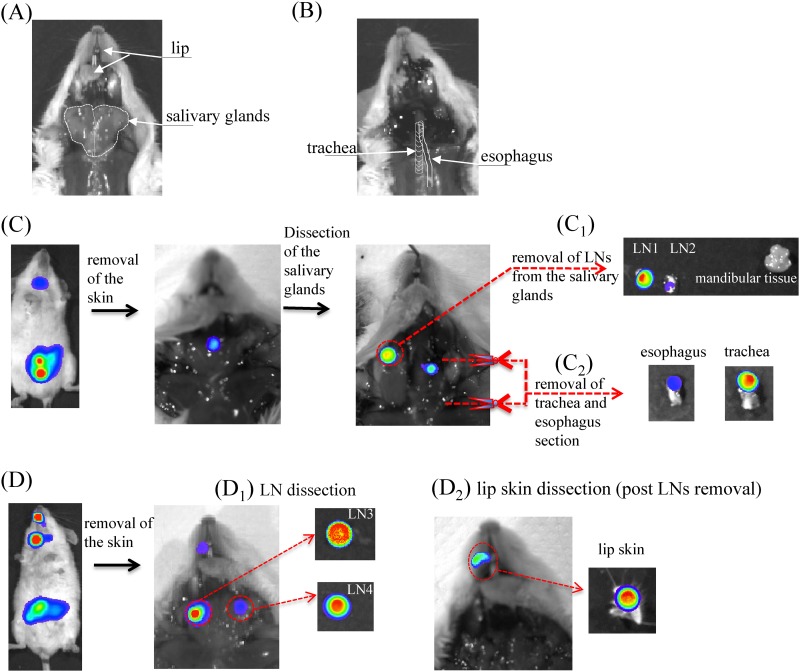
Mouse regional ventral cervical anatomy and analysis of the site of bacterial colonization after needle feeding. (A) Salivary glands outlined in white dotted lines are bilateral and located in the cervical neck. (B) After the removal of salivary glands, the trachea and underneath esophagus are accessible as schematically indicated. (C and D) Mice exhibiting BLI signal in the neck 24 h or 48 h postinfection (needle feeding with 4E8 CFU of IP32953-*lux*) were euthanized using CO_2_, and a dissection procedure associated with BLI acquisition was performed step by step. Two representative mice are shown. After a first step of skin removal, the salivary gland region was dissected, and lymph nodes (LN1 to LN4) found in this region were collected. Then, the esophagus and trachea section were collected as indicated by the schematized red scissors. Regions of interest (ROI) were drawn, and the average bioluminescence (photons/s/cm^2^/sr) was calculated for each dissected organ. Organs exhibiting BLI signal were homogenized in PBS and enumerated for bacterial count (CFU). (C_1_) Lymph nodes LN1, 2E5 ROI, 2E4 CFU; and LN2, 5.4E4 ROI, 4E3 CFU. (C_2_) Esophagus, 2E6 ROI, 4E5 CFU; and trachea, 6E6 ROI, 6E5 CFU. (D_1_) Lymph nodes LN3, 2.5E7 ROI, 4E6 CFU; and LN4, 1E6 ROI, 4E5 CFU. (D_2_) Skin from the mouth, 9.3E5 ROI, CFU not done. Among 10 dissected mice, nine exhibited bioluminescent signals in lymph nodes from the salivary gland region (C_1_ and D_1_), 7 in the esophagus and/or trachea (C_2_), and five close to the mouth/lip region (D_2_).

To identify which specific neck region is colonized by Y. pseudotuberculosis, mice were first orally gavaged with 4E8 CFU of IP32953-*lux*; then, at 24 and 48 h postinfection, mice exhibiting a BLI signal in the neck were euthanized and dissected in the cervical ventral region (see Materials and Methods). After skin removal, all of the mice exhibited a BLI signal coming from the cervical soft tissue composed of salivary glands, lymph nodes (LNs), and adipose tissues (Fig. 2). Among 10 dissected mice, nine exhibited bioluminescent signals in LNs from the salivary gland region (Fig. 2A, C_1_, and D_1_). Although there was no preferential right or left LN colonization, we often noticed that after removal of a first LN producing large amounts of photons, it was possible to identify secondary LNs producing less light, indicating various levels of bacterial colonization among LNs. Each time a BLI-positive LN was isolated, we verified its bacterial content by homogenization and CFU counting on agar plates (Fig. 2C_1_ and D_1_). In addition to LNs, we noticed in 70% of the mice a strong BLI signal in the esophagus and/or trachea (Fig. 2B and C). Dissection of the esophagus and tracheal sections associated with BLI allowed us to localize bacterial colonization at the junction of these two structures (Fig. 2C_2_), suggesting a probable deposition of bacteria in the tissue consecutive to the introduction of the needle in the esophagus. Finally, half of the mice emitted a BLI signal in the oral cavity corresponding most of the time to the skin associated with the lip (Fig. 2D and D_2_). A similar analysis performed on mice infected by needle gavage with the strain Y. enterocolitica WA-*lux* led to the same conclusions, where the BLI signal was detected at the laryngopharynx region, draining LNs and skin from the oral cavity (Fig. S4).

From these results, we speculate that the use of a feeding needle entails a risk of creating lesions in the skin of the mouth as well as in the tissue at the junction of the esophagus and trachea, from which pathogenic *Yersinia* spp. can disseminate to and multiply in the draining LNs located in the salivary gland region.

Although we do not know the impact of the neck colonization by Y. enterocolitica and Y. pseudotuberculosis in the overall infectious process, we cannot exclude the possibility of bacterial dissemination in the bloodstream occurring independently of the intestinal colonization and translocation at the gastrointestinal barrier.

### Bread feeding protects Y. pseudotuberculosis from the acidic gastric environment and promotes efficient intestinal colonization.

**FIG 3:**
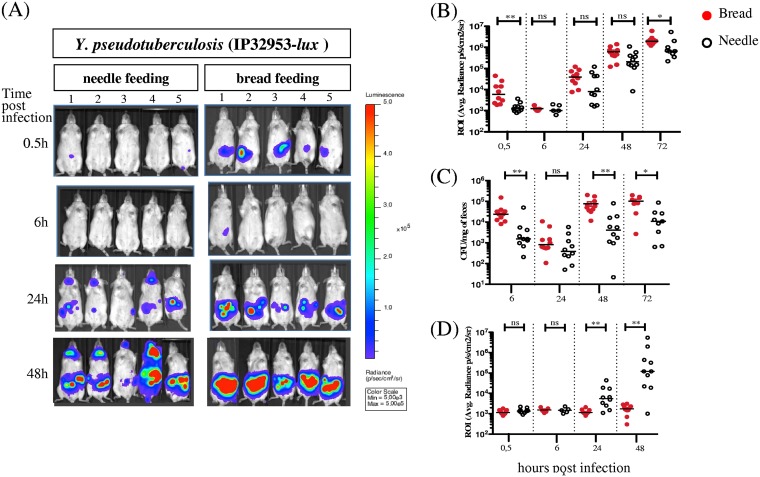
Comparative analysis of bacterial survival upon bread versus needle feeding. OF1 mice were infected with 3.5E8 CFU of IP32953-*lux* Y. pseudotuberculosis using bread or feeding needle, and at 0.5, 6, 24, 48, and 72 h postinfection, mice were imaged using an IVIS Spectrum system. (A) Monitoring of 5 representative mice from 0.5 to 48 h postinfection using needle and bread feeding, same color scale (Min, 5E3; Max, 5E5), with settings of 2 min for exposure time and small binning. (B) ROI were drawn in the abdominal region, and the average bioluminescence (photons/s/cm^2^/sr) was calculated for each mouse at 0.5, 6, 24, 48, and 72 h postinfection using bread (red circle) or needle (open circle). (C) Enumeration of Y. pseudotuberculosis IP32953-*lux* in feces at 0.5, 6, 24, 48, and 72 h postinfection using bread (red circle) or needle (open circle) feeding. (D) ROI were drawn in the neck region, and the average bioluminescence (photons/s/cm^2^/sr) was calculated for each mouse at 0.5, 6, 24, 48, and 72 h postinfection using bread (red circle) or needle (open circle) feeding. The median values are indicated by a horizontal bar. Data were analyzed using the Prism 5 software for nonparametric Mann-Whitney *t* test; **, *P* < 0.002; *, *P* = 0.014; not significant (ns), *P* > 0.05. The increased BLI signal in the abdominal region and bacterial load in the feces when mice are infected by bread feeding indicate a more efficient delivery of bacteria and colonization of the intestinal tract compared to the needle feeding protocol. BLI signal increases over time in the neck of mice infected with the needle, while no signal was detected in the neck of the animals infected with bread.

We next compared the kinetics of bacterial dissemination upon bread feeding versus needle feeding. Mice were orally infected with 3.5E8 CFU of IP32953-*lux* and imaged at 0.5, 6, 24, 48, and 72 h postinfection (Fig. 3). At each time point, photon emission was measured from the abdominal region and neck region. As indicated in Fig. 3A and B, upon bread feeding, the BLI signal in the abdominal region is detected as early as 30 min postinfection; it then decreases to an almost undetectable signal at 6 hours postinfection (h p.i.) and in a second phase increases continuously. Upon needle feeding, the BLI signal is barely detectable before 24 h p.i.; after that time point, the BLI signal is detected in the neck (Fig. 3A and D) and the abdominal region (Fig. 3A and B), where it continuously increases over time. It is noteworthy that the neck region signal was never detected in bread-infected animals (Fig. 3D) even at later time points. Statistical analysis indicates a significantly higher light emission signal in the abdominal region at early (0.5 h) and later (72 h) time points when using bread than when using a needle (Fig. 3B). This observation correlates with the smaller amount of Y. pseudotuberculosis found in the feces 6 h post-needle feeding, as shown by CFU counting (Fig. 3C), suggesting that a lower number of bacteria survive passage through the stomach.

**FIG 4:**
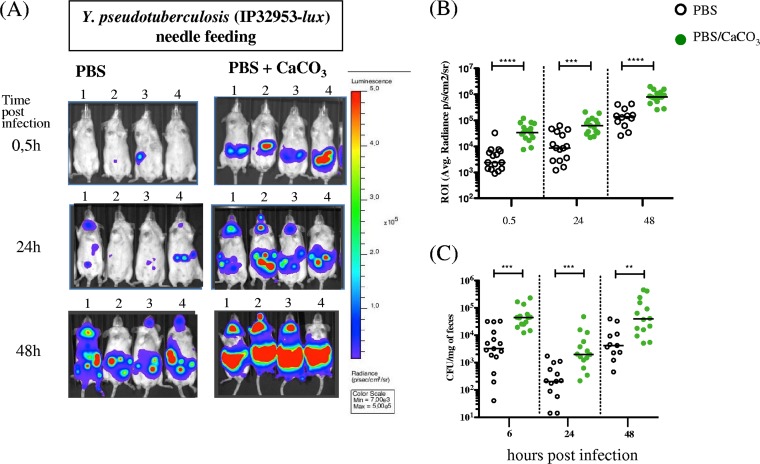
Protective effect of CaCO_3_ when bacteria are administered directly in the stomach by needle feeding. OF1 mice were infected with 4 to 5E8 CFU of Y. pseudotuberculosis IP32953-*lux* using two needle-feeding conditions, bacterial suspension in PBS (open circle) or in PBS supplemented with CaCO_3_ (30 mg/ml) (green circle). At 0.5, 24, and 48 h postinfection, mice were imaged using an IVIS Spectrum system. (A) A representative panel of the animals for the two conditions is shown using the same color scale (min, 7E3; max, 5E5), with settings of 2 min exposure time and small binning. (B) Regions of interest (ROI) were drawn in the abdominal region, and the average bioluminescence (photons/s/cm^2^/sr) was calculated for each mouse. (C) Enumeration of Y. pseudotuberculosis IP32953-*lux* in feces at 6, 24, and 48 h postinfection. The median values are indicated by a horizontal bar. Data were analyzed using the Prism 5 software for nonparametric Mann-Whitney *t* test; ****, *P* < 0.0001; ***, *P* ≥ 0.0001; **, *P* = 0.0014. The addition of CaCO_3_ to the bacterial suspension protects Y. pseudotuberculosis when administered via a feeding needle directly in the stomach.

When needle feeding is used, bacteria are delivered directly to the stomach, a compartment known to have an acidic pH (between 3 and 4) (22) that is aggressive to microorganisms. To evaluate whether the smaller amount of light observed in the abdominal region of needle-infected mice was due to bacterial killing by the acidity of the stomach, we infected animals using the needle or bread with either bacterium resuspended in PBS only or in PBS buffered with CaCO_3_ (23). As shown in Fig. 4A and B, buffering the bacterial inoculating medium significantly increases the BLI signal in the abdominal region, as well as the amount of bacterial CFU recovered in the feces (Fig. 4C) of mice infected with the needle but not with the bread (Fig. S5). Our results therefore indicate that bacterial bread delivery protects Y. pseudotuberculosis from the acidic gastric environment and promotes efficient intestinal colonization without the requirement of an additional buffering compound.

### Bread feeding is a more physiological oral administration method to study Y. pseudotuberculosis infection.

**FIG 5:**
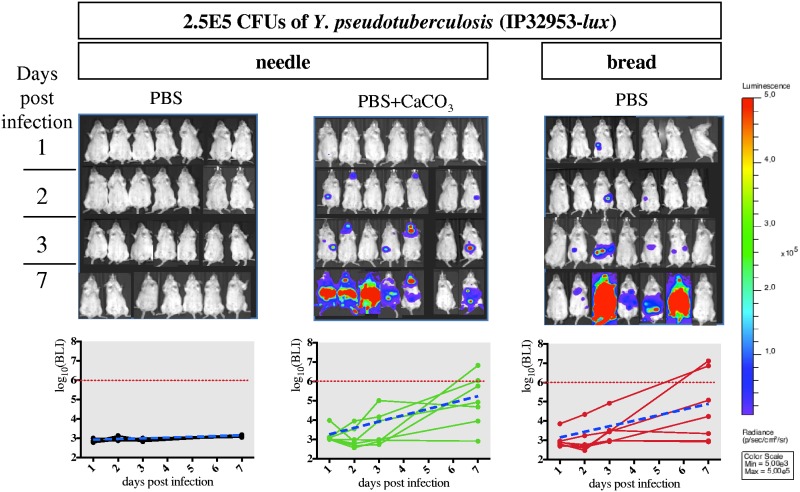
Comparative analysis of the course of infection using the three protocols of administration with 2.5E5 CFU of Y. pseudotuberculosis. OF1 mice were orally infected with 2.5E5 CFU of Y. pseudotuberculosis strain IP32953-*lux* and monitored over time for BLI signal after needle feeding of bacteria resuspended in PBS (black), in PBS supplemented with CaCO_3_ (green), or after bread feeding of bacteria resuspended in PBS only (red). Quantification of the bioluminescent signal from the abdominal region for each individual mouse is shown below the mouse pictures under each condition at each time point (1, 2, 3 and 7 days p.i.). A linear mixed model was used to test for the differences in the BLI signal evolution (on the log_10_ scale) between the three protocols of administration. A mean slope indicated as a blue dotted line of the BLI signal evolution was calculated with the linear mixed model for each protocol. The comparative analysis of the mean slopes indicates significant statistical differences in the BLI signal evolution between needle feeding with bacteria in PBS supplemented with CaCO_3_ and needle feeding with bacteria in PBS only (*P* = 0.012), and between bread and needle feeding with bacteria in PBS only (*P* = 0.03). Red dotted lines indicate the average radiance of ROI measurements above which animals are in terminal illness. Bread feeding allows an efficient infection of the abdominal region by Y. pseudotuberculosis without neck colonization compared to the needle feeding.

**FIG 6:**
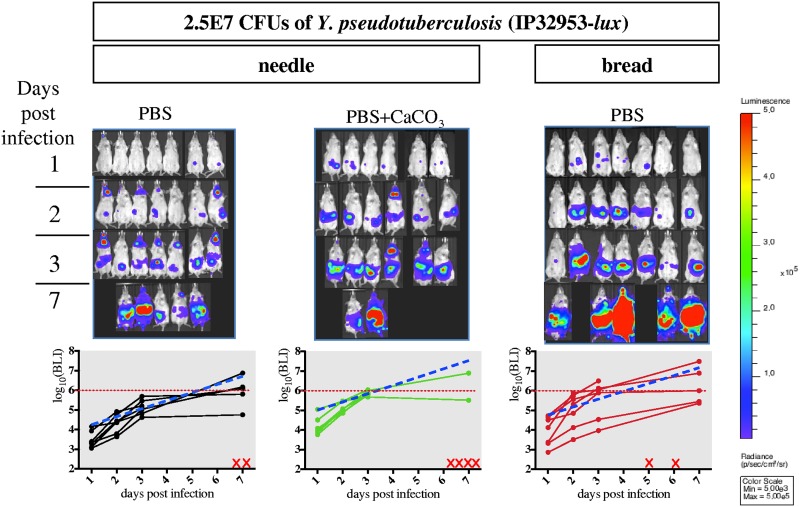
Comparative analysis of the course of infection using the three protocols of administration with 2.5E7 CFU of Y. pseudotuberculosis. OF1 mice were orally infected with 2.5E7 CFU of Y. pseudotuberculosis strain IP32953-*lux* and monitored over time for BLI signal after needle feeding of bacteria resuspended in PBS (black line), in PBS supplemented with CaCO_3_ (green line), or after bread feeding of bacteria resuspended in PBS only (red line). Abdominal region BLI imaging (top) and quantification (bottom) at each time point (1, 2, 3, and 7 days p.i.) for each individual animal are shown. A linear mixed model was used to test for the differences in the BLI signal evolution (on the log_10_ scale) between the three protocols of administration. A mean slope indicated as a blue dotted line of the BLI signal evolution was calculated with the linear mixed model for each protocol. The comparative analysis of the mean slopes indicates no significant statistical differences in the BLI signal evolution between the three modes of bacterial administration (*P* > 0.05). Red dotted lines indicate the average radiance of ROI measurements, above which animals are in terminal illness. Red crosses positioned on the *x* axis indicate dead mice.

The better survival of Y. pseudotuberculosis when associated with CaCO_3_ prior to needle feeding led us to evaluate whether it would change the overall 50% lethal dose (LD_50_) of strain IP32953-*lux* when administered with bread. Thus, mice were infected using three conditions (needle with or without supplementation of CaCO_3_ and bread) with four different Y. pseudotuberculosis IP32953-*lux* concentrations (2.5E5, 2.5E6, 2.5E7, and 2.5E8 CFU). The doses were chosen where the lowest and the highest doses are expected to kill 0% and 100% of the mice, respectively. Animals were monitored for lethality up to 21 days postinfection to allow measurements of LD_50_. The use of the needle without supplementation of CaCO_3_ gave the highest LD_50_ (5.2E6 CFU). When bacteria are mixed with CaCO_3_, the LD_50_ is lower (1.8E6 CFU), suggesting a protective effect of CaCO_3_ when the needle-feeding administration is used. However, the lowest calculated LD_50_ was obtained when mice were infected with bread (LD_50_, 9.5E5). Even though the statistical comparison (log rank test) of the mouse survival did not reveal significant differences among the three bacterial administration routes (Fig. S6), measurement of the abdominal BLI signal at the lowest infectious dose (2.5E5 bacteria, Fig. 5) shows a clear difference between needle PBS (no increase in the abdominal signal) versus bread and needle PBS plus CaCO_3_ treatments (significant increase in the abdominal signal). Interestingly, this difference in the increase of the abdominal BLI signal between treatments is not observed any more at a higher infectious dose (2.5E7, Fig. 6), suggesting that the bread and the needle PBS plus CaCO_3_ treatments allow a more sensitive monitoring of bacterial infection progression at low infectious doses (Fig. S6). It is noteworthy that even at low bacterial concentrations (2.5E5 CFU), needle feeding (with or without CaCO_3_) leads to neck colonization, whereas none of the mice showed a neck BLI signal upon bread feeding (Fig. 5). Our results therefore suggest that infection using bread feeding is more physiological for Y. pseudotuberculosis delivery to the intestinal tract, resulting in an efficient colonization even at low inoculum concentrations.

## DISCUSSION

When studying host pathogen interactions, the choice of a laboratory experimental model of infection is crucial. Besides the choice of the animal species and the pathogen to study, important parameters that have to be taken into account include the control of the dose of the infectious agent administered to each animal, the rapidity to handle the animal, and the stress induced by the animal manipulation. For decades, laboratories interested in studying animal oral infection have used needle feeding to orally deliver infectious agents. Although needle feeding allows control of the delivered dose, there are drawbacks, such as the requirement of a specific manipulator training, the use of anesthesia when animals are particularly agitated, or the stress induced by the handling of the animal. In the present work, we show that bread feeding allows a nontraumatic pathogen administration, where animal handling is minimized and therefore is less stressful for both the manipulator and the animal. We found that the habituation step to feed on bread few days prior to the infection is crucial for effective bread feeding, with no need of additional melted butter as a vehicle as proposed by others (7, 8). Bread feeding constitutes a very good administration method since it allows control of the dose administered and is a fast procedure, where all bread is generally consumed by mice in less than 5 min, as opposed to the drinking water method (within several hours) described by others (10).

Importantly, in this study, we demonstrate that the use of needle gavage induces damage to the oral cavity of infected animals, leading to pathogen colonization in tissues and organs distant from the intestinal tract. Although it is not known whether such artificial colonization affects the intestinal infection process, it is reasonable to question its impact on the overall host immune response. For example, Barnes and collaborators have reported Y. pseudotuberculosis translocation to organs such as the liver and spleen shortly after oral inoculation with a feeding needle (16). Although the authors did not mention neck injury, it is possible that some lesions caused by the needle participated in this early translocation. In this context, it is important to mention that our results suggesting no significant differences in the LD_50_ between mice infected via needle versus bread feeding may potentially mask important differences in organ colonization.

Our study also shows that bacteria directly delivered in PBS via needle feeding are susceptible to the acidic environment of the stomach. Even though this susceptibility can be rescued by buffering the bacterial suspension with CaCO_3_, this infection method still produces artificial neck colonization. On the contrary, bread feeding provides bacterial protection against the stomach acidic environment and fully eliminates colonization of the neck region. Overall, our results encourage us to reinvestigate the infectious process by Y. pseudotuberculosis and Y. enterocolitica employing bread feeding, a very robust and more physiological administration method that can be potentially extended to other enteropathogen infection models.

## MATERIALS AND METHODS

### Culture conditions and bacterial strain construction.

Bacteria were grown at 28°C in lysogeny broth (LB) or on LB agar (LBA) plates. For recombinant bacterial growth, control strains were also grown at 28°C in minimal medium containing 1× M9 salts, 2 mM MgSO_4_, 0,1 mM CaCl_2_, 1% glucose, and 1% Casamino Acids. Growth of parental and recombinant *lux* strains in liquid culture was analyzed by diluting overnight cultures to fresh medium after being washed three times in PBS when minimum medium was used. Bacterial concentrations were evaluated by spectrometry at 600 nm and plating on LBA. Kanamycin (Km; 30 μg/ml) or Irgasan (0.1 μg/ml) was added to the media when necessary.

The fully virulent serotype 1b Y. pseudotuberculosis IP32953 strain, isolated from a human stool sample in France, and the biotype 1B serotype O:8 Y. enterocolitica WA strain, isolated from the blood of a human patient, were used as parental strains to generate their bioluminescence-expressing derivative strains, IP32953-*lux* and WA-*lux*, respectively. As previously described (21), the Photorhabdus luminescens *luxCDABE* operon controlled by the *rplN* constitutive *Yersinia* promoter was introduced into the *Yersinia* chromosome via the Tn*7* technology (20). Plasmid pUCR6K-mini-Tn*7-Km^r^-luxCDABE* and the transposase-encoding plasmid pTNS2 were coelectroporated to IP32953 or conjugated to WA cells. Bioluminescent recombinant clones were selected on LB agar plates supplemented with Km. The recombinant IP32953-*lux* and WA-*lux* strains were verified for (i) Tn*7*-P*rplN*-lux chromosomal insertion by PCR using primers flanking the Tn*7* insertion site (P*glmS* [5′-GCTATACGTGTTTGCTGATCAAGATG-3′], P*pstS* [5′-ACGCCACCGGAAGAACCGATACCT-3′], PTn*7*L [5′-ATTAGCTTACGACGCTACACCC-3′], and PTn*7*R [5′-CACAGCATAACTGGACTGATTTC-3′]), (ii) a growth rate similar to that of the parental strain in LB, and (iii) similar virulence via the oral route by bread feeding administration. The stability of the *lux* operon was verified by successive subcultures in LB without antibiotic pressure and measurements of the BLI signal on CFU resuspended in 0.1 ml LB using a Xenius 96-well plate reader (SAFAS Monaco).

### Ethics statement.

Animals were housed in the Institut Pasteur animal facility accredited by the French Ministry of Agriculture to perform experiments on live mice (accreditation B 75 15-01, issued on 22 May 2008), in compliance with French and European regulations on the care and protection of laboratory animals (EC Directive 86/609, French Law 2001-486 issued on 6 June 2001). The research protocol was approved by the French Ministry of Research (no. CETEA 2014-0025) and Institut Pasteur CHSCT (no. 0399).

### Animal experiments.

Female 7-week-old OF1 mice were purchased from Charles River France and allowed to acclimate for 1 week before infection. Prior to all infections (bread or needle), mice were fasted for 16 h and had continuous access to water. Only bread-fed mice were trained to feed on bread prior to the infection. All oral infections were performed on nonanesthetized animals. Serial dilution of bacterial suspensions was performed in phosphate-buffered saline (PBS without CaCl_2_ and MgCl_2_) from cultures grown for 48 h at 28°C in LB agar plates.

For oral gavage, mice were administered a 0.2-ml bacterial suspension using an animal feeding stainless steel bulbous-ended needle (0.9 mm by 38 mm, 20 G by 1.5”, catalog no. 9921; Cadence Science). The bulbous-ended needle was inserted over the tongue into the esophagus and stomach, as previously described (23). When required, the 0.2-ml bacterial suspension used for oral gavage (feeding needle) was mixed with 0.3 ml of a 50-mg ml^−1^ suspension of CaCO_3_ in PBS without CaCl_2_ and MgCl_2_, and the 0.5-ml mixture was administered. Since CaCO_3_ is not soluble at this concentration, the bacterial suspension was mixed with CaCO_3_ at once before each feeding-needle injection.

For bread feeding, mice were first adapted to feed on bread prior to the infection. Thus, 3 days before infection, the food was replaced by small pieces of white bread (approximately 9 mm^2^) to allow mice to feed on bread for a 2-h period. The same bread adaptation was repeated once 24 h before infection. Then, 16 h prior to the infection, the food was removed, and mice were fasted with access to water. A 20-μl bacterial suspension (with or without supplementation of CaCO_3_) was deposited on one piece of bread and placed in an empty and clean cage, where one mouse was introduced. Each mouse was visually monitored until complete bread feeding. Generally, bread feeding took from 30 s up to 10 min per mouse. After feeding, animals were housed in a cage with new litter and access to food and water *ad libitum*.

After infection, animals were monitored daily for 21 days, and every day, the litter was renewed in order to limit accumulation of feces in the cage and avoid cross-contamination between mice.

### BLI imaging and dissection.

*In vivo* imaging was performed with the *In Vivo* Imaging System (IVIS 100; Caliper Life Sciences). Animals were anesthetized using a constant flow of 2.5% isoflurane mixed with oxygen. Images were acquired with binning of 4 and an exposure time from 10 s to 2 min, according to the signal intensity. To quantify the luminescence signal, regions of interest (ROI) were drawn, and measurements of the ROI are given as average radiance (photons/s/cm^2^/steradian [sr]). Uninfected mice were used to set the light emission background.

Analysis of the cervical region was performed sequentially starting with the removal of the skin on euthanized animals and imaging, followed by removal of the most intense bioluminescent tissue and imaging again of the animal to identify a lower intensity signal. When signal was still detected after removal of the first identified bioluminescent tissue, the same sequence was repeated until no signal could be detected. Organs and tissues were aseptically removed, placed in glass beads containing tubes of 0.5 ml PBS, and subjected to homogenization to determine bacterial loads. Feces were collected from live mice and were homogenized in PBS using disposable homogenizers (Kimble Chase piston pellet; Fisher Scientific), and serial dilutions were performed to determine bacterial loads.

### Statistical analysis and LD_50_ calculation.

Statistical analyses were performed with nonparametric Mann-Whitney *t* test and with the GraphPad Prism 5.0 software (San Diego, CA, USA). For comparison of BLI signals within the same animal, data were analyzed with a nonparametric Wilcoxon matched-pairs signed-rank test. Survival curves were compared two by two using a log rank (mantel-Cox) test. A linear mixed model was fitted using the nlme R package to test for the difference of the BLI signal evolution (on the log_10_ scale) between the three protocols of administration (bread feeding or needle feeding with or without CaCO_3_). The mouse effect was included as random so that a random intercept and slope were attributed for each animal. The calculated mean slopes were compared, and *P* values of ≤0.05 were considered significant.

To determine the 50% lethal dose (LD_50_), mice (six to seven per dose) were infected with 10-fold serial dilutions of bacterial suspensions (from 2E5 to 2E8 CFU) and were monitored for 3 weeks. The LD_50_ was calculated using the Reed and Muench method (24), where the survival (*y*_1_; …; *y*_i_; … ; *y*_n_) and death (*z*_1_; … ; *z_i_*; … ; *z_n_*) numbers for each dose (*x*_1_; … ; *x_i_*; … ; *x_n_*) are measured. The method takes into account the cumulative values of death (for dose *p*, $Y_{p}=\overset{p}{\underset{i=1}{\sum }}y_{i}$) and survival (for dose *p*, $Z_{p}=\overset{n}{\underset{i=p}{\sum }}z_{i}$) for all the doses. These values allow the calculation of mortality as *M_p_* = *Y_p_*/(*Z_p_* + *Y_p_*). For *M_p_* of 0.5 (the mortality at the 50% lethal dose), we chose the dose $x_{j}$ as *M_j_* ≤ 0.5 < *M_j_*_+1_, and then we calculated the proportional distance (D) as (0.5 – *M_j_*)/*M_j_*_+1_ – *M_j_*), LD_50_ = $10^{\log(x_{j})+D}$.

## Supplementary Material

Supplemental file 1

Supplemental file 2

Supplemental file 3

Supplemental file 4

Supplemental file 5

Supplemental file 6

Supplemental file 7
