# Supplementary figures and images for: Bread Feeding Is a Robust and More Physiological Enteropathogen Administration Method Compared to Oral Gavage
Source: Infect Immun. 2020 Mar 23;88(4):e00810-19. doi: 10.1128/IAI.00810-19 (PMC7093149; doi:10.1128/IAI.00810-19)

Fig. S1

(A)

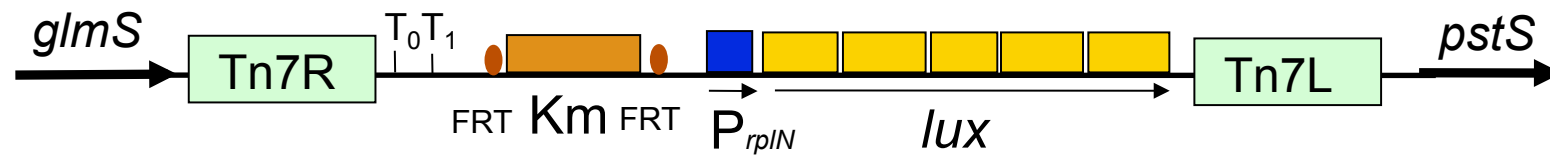

Strain *Y. pseudotuberculosis* = IP32953::Tn7-PrpIN-lux

(B)

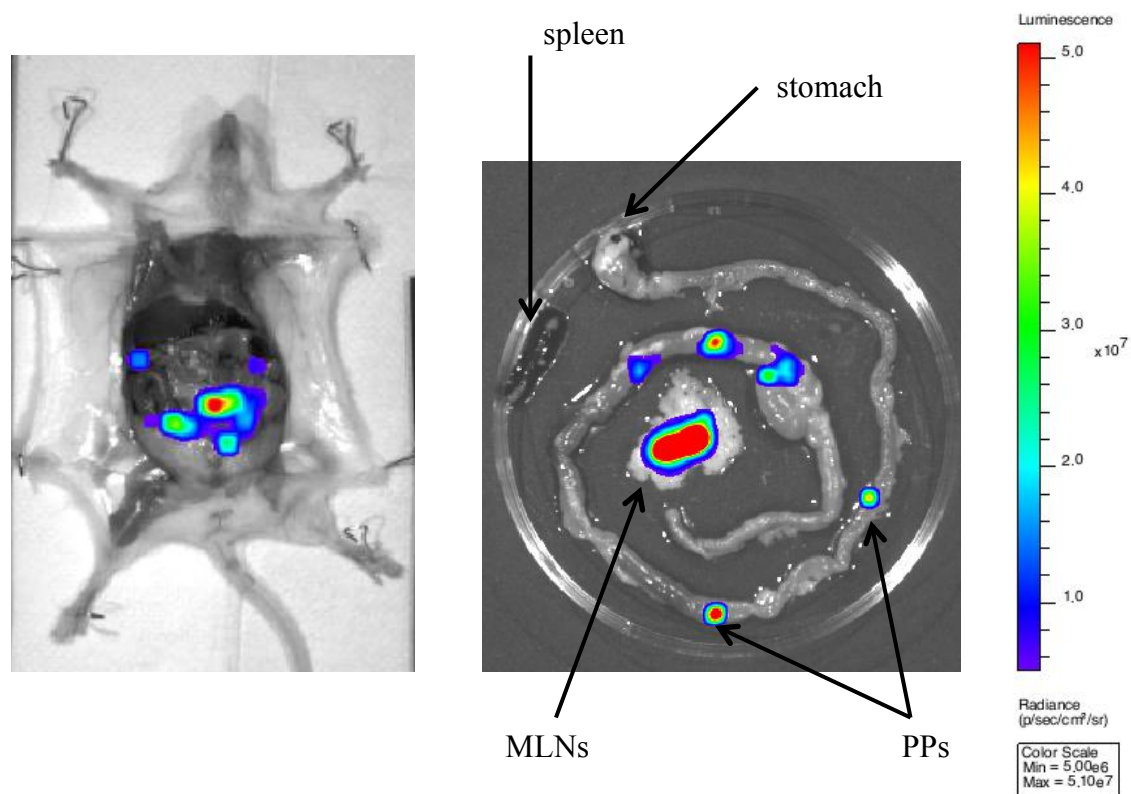

Supplement: Supplemental file 1 [file IAI.00810-19-s0001.pdf]

Fig. S2

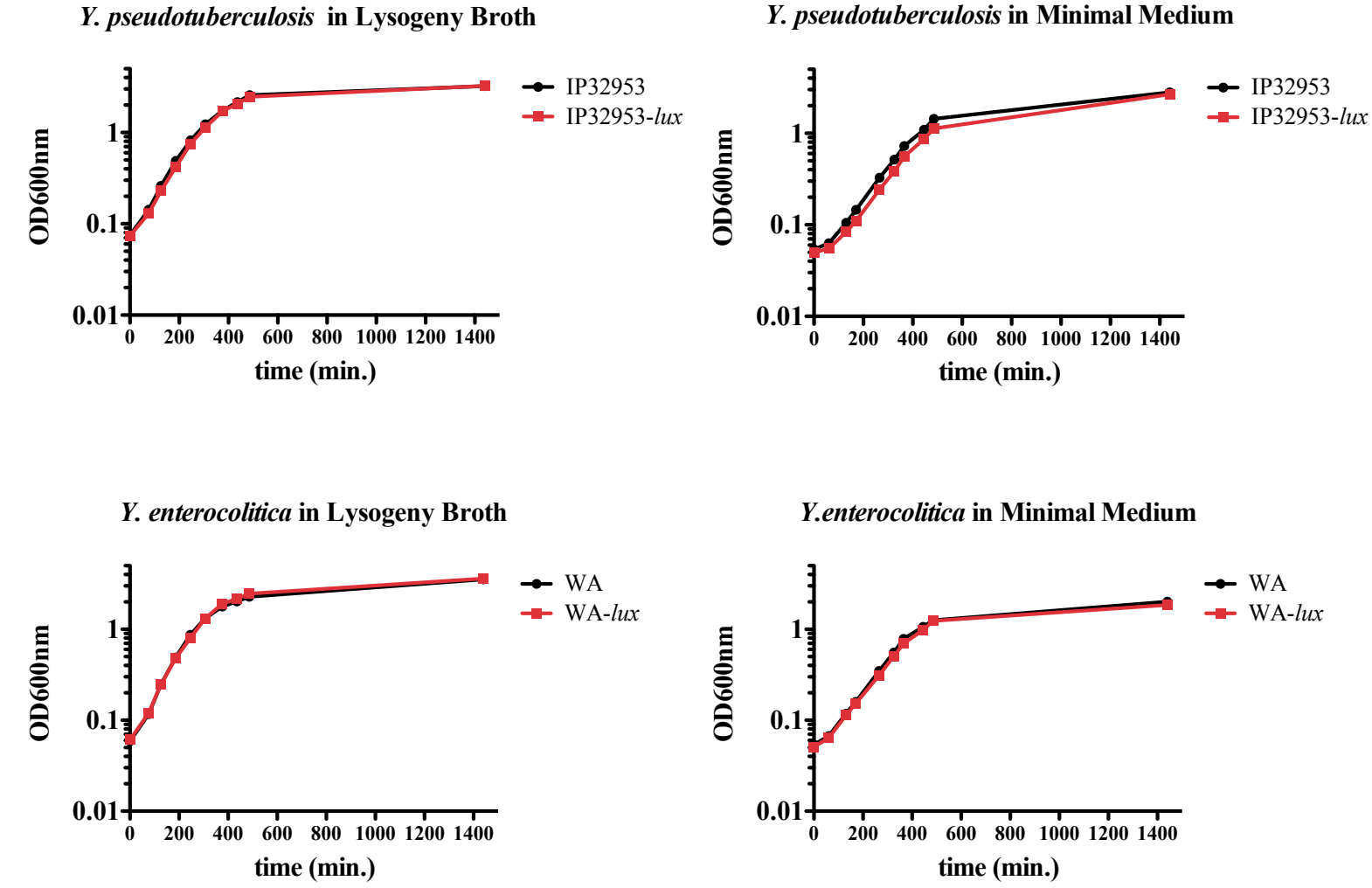

Supplement: Supplemental file 2 [file IAI.00810-19-s0002.pdf]

**Fig. S3**

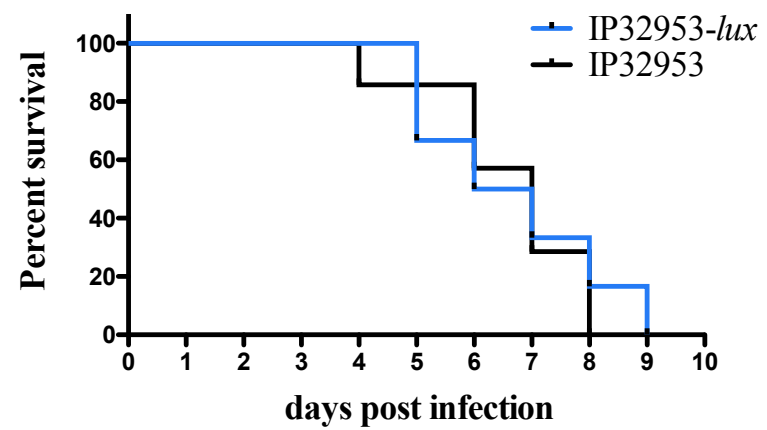

Supplement: Supplemental file 3 [file IAI.00810-19-s0003.pdf]

Fig. S4

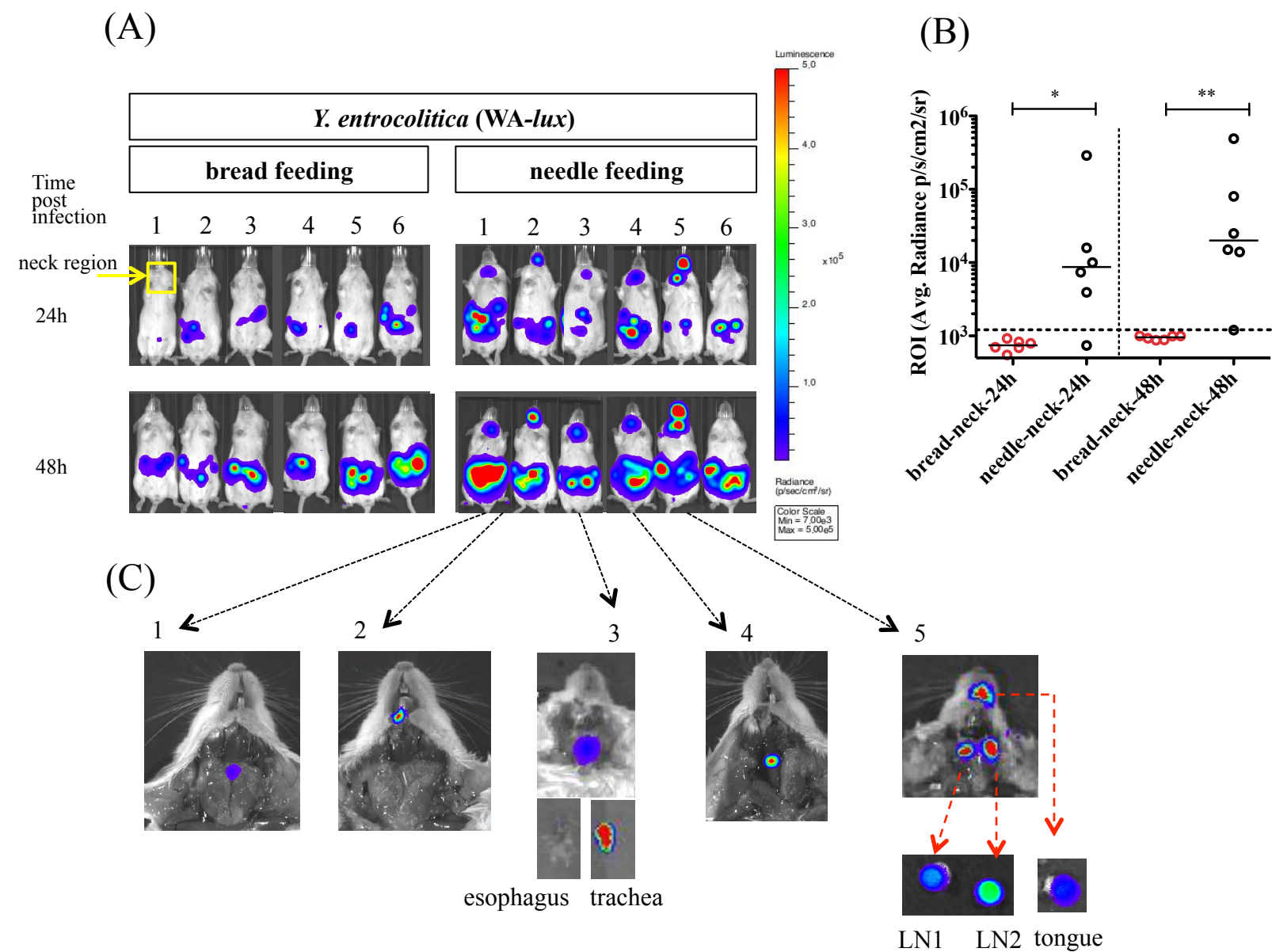

Supplement: Supplemental file 4 [file IAI.00810-19-s0004.pdf]

Fig. S5

(A)

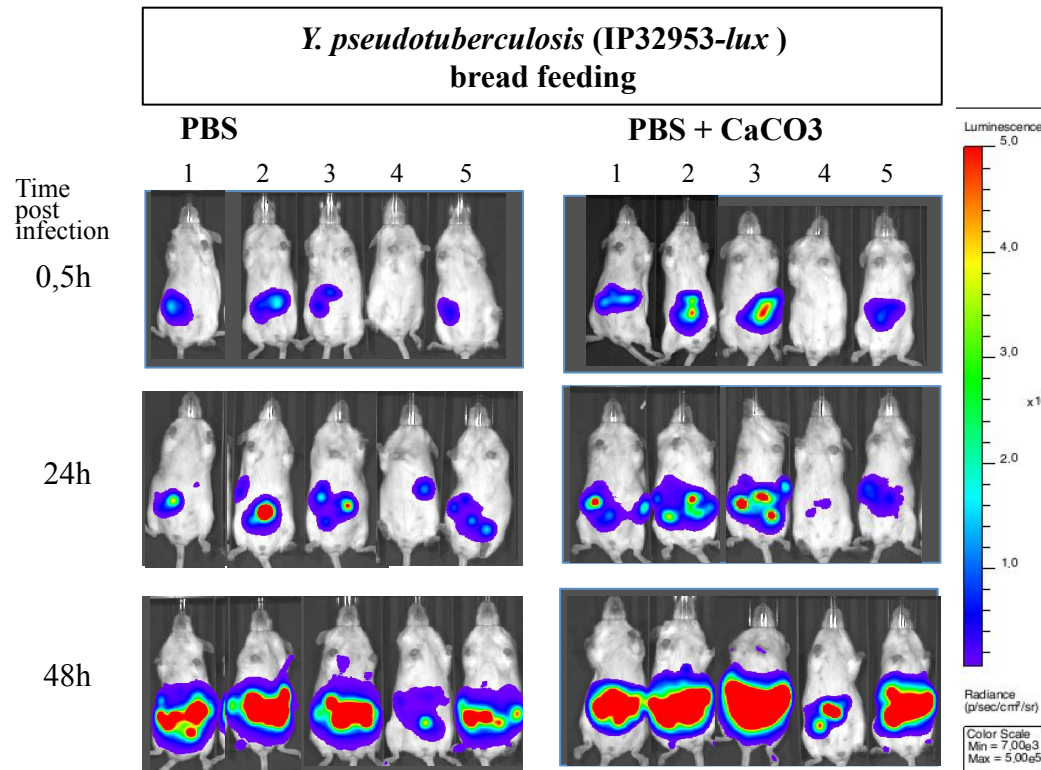

(B)

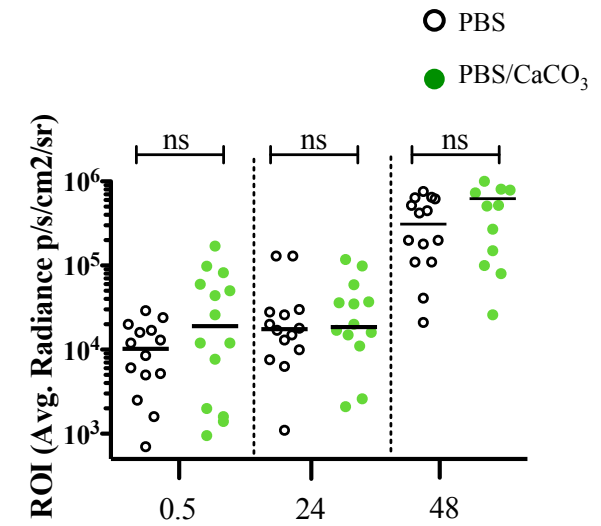

(C)

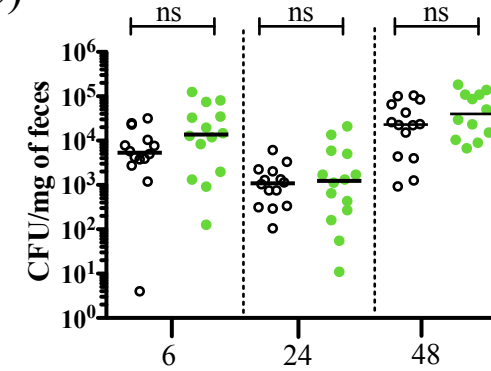

Supplement: Supplemental file 5 [file IAI.00810-19-s0005.pdf]

**Fig. S6**

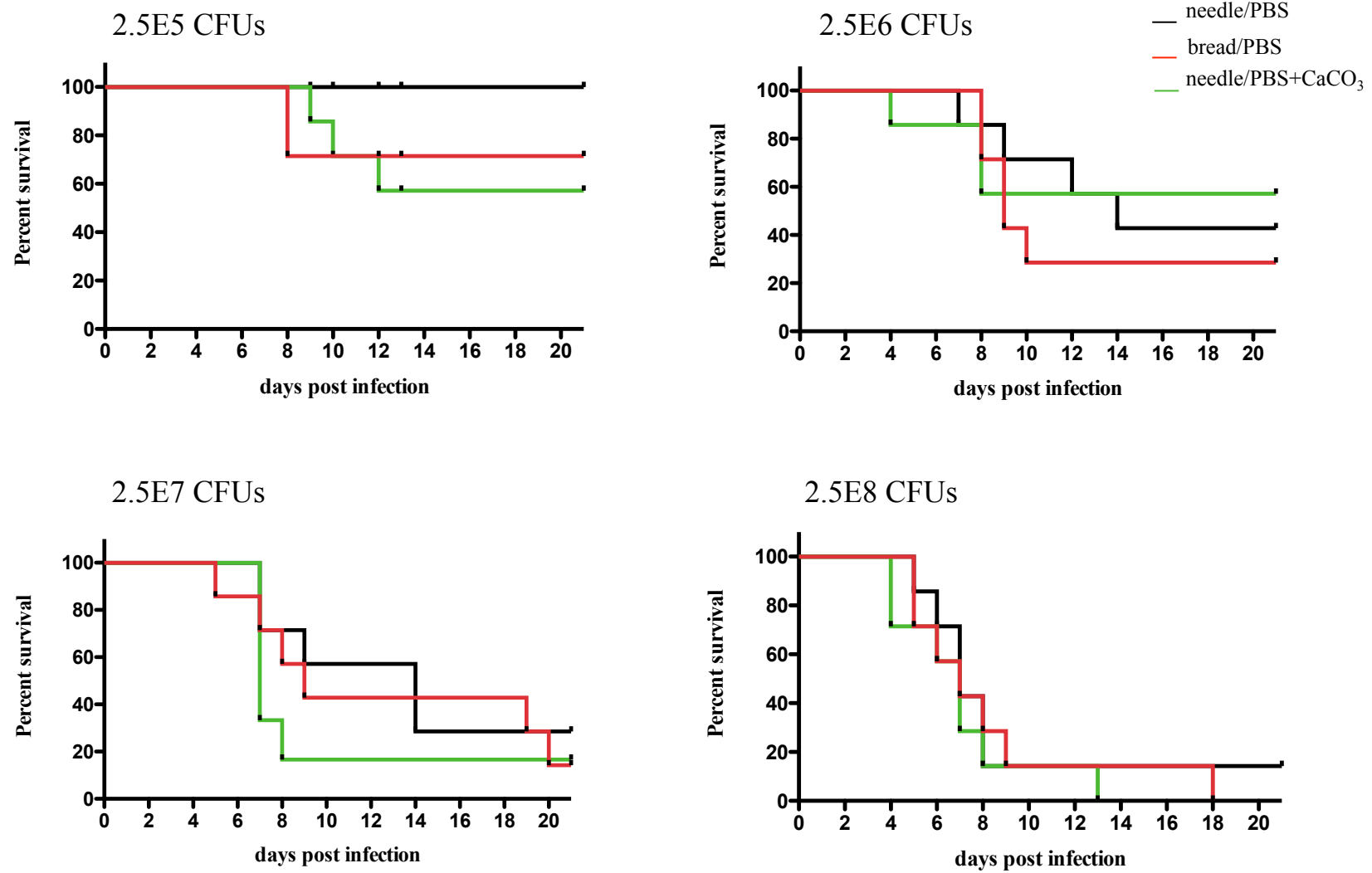

Supplement: Supplemental file 6 [file IAI.00810-19-s0006.pdf]
