# Supplementary material for: Bread Feeding Is a Robust and More Physiological Enteropathogen Administration Method Compared to Oral Gavage
Source: Infect Immun. 2020 Mar 23;88(4):e00810-19. doi: 10.1128/IAI.00810-19 (PMC7093149; doi:10.1128/IAI.00810-19)
Supplement: Supplemental file 7 [file IAI.00810-19-s0007.pdf]

Legends for supplemental material

Fig. S1 (A) genetic map of the mini *Tn7-Km-PrplN-lux* integrated in the chromosome of IP32953. (B) Pilot experiment to confirm the translocation of *Y. pseudotuberculosis* IP32953-*lux* to Peyers' patches (PP) and mesenteric lymph nodes (MLN) after bread feeding with 0.8E8 bacterial CFUs. At day 4 p.i. the mouse was euthanized with CO_2 and dissected to confirm intestinal infection by BLI measurement.

Fig. S2 Growth curves of *Y. pseudotuberculosis-lux* and *Y. enterocolitica-lux* and their respective parental strain. Bacteria were grown at 28°C under agitation in 50 mL lysogeny broth or minimal medium and optical absorbance was followed for 24 hours. Both bioluminescent recombinants grow similarly to their parental strains.

Fig. S3 Comparative survival curve of *Y. pseudotuberculosis-lux* and its parental strain. OF1 mice were bread fed with 4E8 CFU of either IP32953-*lux* or IP32953. Mice were observed daily and survival was recorded. Data were analyzed using Prism 5 software for Log-rank (mantel-Cox) test. Both strains killed all mice (n=7 per group) with the same kinetic.

Fig. S4 Comparative bioluminescence imaging of mice infected by either needle or bread feeding with *Y. enterocolitica*.

OF1 mice were infected with 2E9 CFUs of *Y. enterocolitica* bioluminescent strain (WA-*lux*) using a 20G x 1.5” feeding needle or a piece of bread. (A) At 24 and 48 hours (h) post infection mice were imaged using an IVIS Spectrum imaging system with an acquisition time of 2 minutes and small binning. Uninfected mice (UI) were used to set the light emission background. Regions of interest (ROI) were drawn in the neck region (yellow frame) using the Living Image 4.5 software. (B) ROI average bioluminescence (photon/sec/cm2/sr) was

calculated for each individual mouse infected by needle gavage (in black) or by bread feeding (in red). The horizontal doted line indicates the light emission background. Data were analyzed using Prism 5.0 software for T test non-parametric Mann Whitney, p=0,015(*), p=0,005(**). Median of the values is indicated by a horizontal bar. Bioluminescence signal is detected in the neck of 83% of the needle infected animals, whereas none of the mice infected with bread presented a signal in the neck. (C) Mice exhibiting BLI signal in the neck 48 h post-infection were euthanized and dissected to localize the site of infection. Bioluminescent *Y. enterocolitica* was found in the trachea, draining lymph nodes and tongue.

Fig. S5 Evaluation of the effect of $CaCO_3$ when bacteria are administered via bread feeding.

OF1 mice were infected with 1.6E8 *Y. pseudotuberculosis* IP32953-*lux* CFUs using two conditions (14 mice per condition): bacterial suspension in PBS (open circle) or in PBS supplemented with $CaCO_3$ (30 mg/ml) (green circle). At 0.5, 24 and 48 h post infection mice were imaged using an IVIS Spectrum imaging system. (A) A representative panel of the animals for the two conditions are shown using same color scale (min= 7E3 max= 5E5) with settings 2min time of exposure and small binning. (B) Regions of interest (ROI) were drawn in the abdominal region and average bioluminescence (photon/sec/cm2/sr) was calculated for each mouse. (C) Enumeration of *Y. pseudotuberculosis* IP32953-*lux* in feces at 6, 24, and 48 h post infection. Median of the values is indicated by a horizontal bar. Data were analyzed using Prism 5 software for T test non-parametric Mann Whitney, p>0,05 (ns), Addition of $CaCO_3$ to the bacterial suspension had no significant impact on bacterial colonization of mice after bread feeding.

Fig. S6 Comparative analysis of animal survival after oral infection with *Y. pseudotuberculosis*.

OF1 mice survival after oral infection with serial dilutions of *Y. pseudotuberculosis* IP32953-*lux* using bread feeding (red line), needle feeding supplemented with $CaCO_3$ (green line) or needle feeding without $CaCO_3$ (black line). Mice were observed daily for 21 days. Data were analyzed using Prism 5 software and survival curves were compared two by two using a Log-rank (mantel-Cox) test. No statistical significant differences were calculated between the different treatments.
